# The Potential Effects of Oxidative Stress-Related Plasma Abnormal Protein Aggregate Levels on Brain Volume and Its Neuropsychiatric Consequences in Parkinson's Disease

**DOI:** 10.1155/2021/3666327

**Published:** 2021-08-14

**Authors:** Pei-Chin Chen, Chiun-Chieh Yu, Yueh-Sheng Chen, Cheng-Hsien Lu, Shan-Ho Chan, Kun-Hsien Chou, Mei-Hsiu Chen, Meng-Hsiang Chen, Wei-Che Lin

**Affiliations:** ^1^Department of Diagnostic Radiology, Kaohsiung Chang Gung Memorial Hospital, Kaohsiung, Taiwan; ^2^Chang Gung University College of Medicine, Kaohsiung, Taiwan; ^3^Department of Neurology, Kaohsiung Chang Gung Memorial Hospital, Kaohsiung, Taiwan; ^4^Department of Medical Imaging and Radiology, Shu-Zen Junior College of Medicine and Management, Kaohsiung, Taiwan; ^5^Brain Research Center, National Yang Ming Chiao Tung University, Taipei, Taiwan; ^6^Institute of Neuroscience, National Yang Ming Chiao Tung University, Taipei, Taiwan; ^7^Department of Beauty Science, Meiho University, Taiwan

## Abstract

**Background:**

Oxidative stress has been implicated in the pathogenesis of many diseases, including Parkinson's disease. Large protein aggregates may be produced after the breakdown of the proteostasis network due to overt oxidative stress. Meanwhile, brain volume loss and neuropsychiatric deficits are common comorbidities in Parkinson's disease patients. In this study, we applied a mediation model to determine the potential influences of oxidative stress-related plasma abnormal protein aggregate levels on brain volume and neuropsychiatric consequences in Parkinson's disease.

**Method:**

31 patients with PD and 24 healthy controls participated in this study. The PD patients were further grouped according to the presentation of cognitive decline or not. All participants received complete examinations to determine plasma abnormal protein aggregates levels, brain volume, and neuropsychiatric performance. The results were collected and analyzed in a single-level three-variable mediation model.

**Results:**

Patients with PD cognitive decline exhibited higher plasma NfL levels, decreased regional brain volume, and poor neuropsychiatric subtest results compared with PD patients with normal cognition, with several correlations among these clinical presentations. The mediation model showed that the superior temporal gyrus completely mediated the effects of elevated plasma NfL levels due to the poor psychiatric performance of picture completion and digit span.

**Conclusion:**

This study provides insight into the effects of oxidative stress-related plasma abnormal protein aggregate levels on regional brain volume and neuropsychiatric consequences in Parkinson's disease patients.

## 1. Introduction

### 1.1. Oxidative Stress and Misfolding Protein

Oxidative stress plays a significant role in the pathogenesis of many diseases, including neurodegenerative diseases [1]. Previous studies have suggested that the overproduction of reactive oxygen species and nitrogen species derived from high NADPH oxidase 2 activation could causing oxidative stress [2] and may play complex roles in facilitating the disease development [3]. In particular, evidence of increased lipid peroxidation in the brain of Parkinson's disease (PD) patients has also been proposed [4]. The polyunsaturated fatty acids in the brain, including docosahexaenoic acid and arachidonic acid, are vulnerable to lipid peroxidation owing to the highly unsaturated bonds of their respective structures [5]. The chain reactions of free radical-mediated injury caused by lipid peroxidation could irreversibly oxidize the proteins and influence the proteostasis network [6]. Finally, the maintenance of proteostasis may deteriorate in association with aging and repeated oxidative stress attacks, and then, many large protein aggregates, including neurofilament light chain, A*β* peptide, T-tau, and *α*-synuclein, are produced and accumulation threatens neurons and exacerbates neurodegeneration.

### 1.2. Advanced Technique for Plasma Misfolding Protein Level Evaluation

Traditionally, body fluid biomarkers, including cerebrospinal fluid (CSF), serum, or plasma, have been evaluated by immunoassay techniques. However, the accuracy of the results may be affected owing to the manual protocols involved, indicating the need for a more objective and precise method. The immunomagnetic reduction-based immunoassay (IMR) was introduced, and the application was validated to quantitatively measure the plasma A*β* peptide at ultralow concentrations in Alzheimer's disease patients [7]. Subsequently, the application of IMR for the measurement of plasma tau and *α*-synuclein for the investigation of cognitive impairment in PD patients was reported [8, 9]. More recently, the discrimination of PD patients from normal controls was performed via measuring the plasma neurofilament light chain (NfL) by IMR [10]. Owing to the establishment of IMR sensitivity for the measurement of blood plasma protein aggregates, more precise results of these abnormal protein aggregates in plasma could be determined, and possible correlations with clinical data or potential pathophysiology could be indicated in PD cognitive decline.

### 1.3. Vulnerable Regional Brain Volume Loss and Cognitive Decline

Studies have reported alterations of both dopaminergic and nondopaminergic transmitter systems in the PD brain with different degrees of cognitive impairment [11]. Vulnerable brain regions associated with different network changes affect various types of cognitive decline in PD patients, while different network connectivity changes result in various types of cognitive impairment in PD patients [12, 13]. Progressive cognitive decline in PD is associated with altered function in multiple brain regions [14] and disruptions of dopamine-dependent transmitter systems cause various brain network alterations in PD patients [15]. Furthermore, a previous review study indicated cognitive deficits associated with temporal lobe atrophy, further damaging the networks associated with cognitive decline [16]. The results of these previous studies led us to utilize the voxel-based morphology (VBM) to bridge the possible relationship between plasma abnormal protein aggregates levels and cognitive deficits.

### 1.4. Mediations

Based on previous reports, the oxidative stress microenvironment affecting neurons could result in not only the generation of abnormal protein aggregates but also deficits in the microstructure of the brain, and possibly brain volume loss or neuron loss [1, 17]. Additionally, if vulnerable brain regions play important roles in cognitive function, poor performance in neuropsychiatric tasks could be expected. These clues indicate the possibility of a causal relationship between alterations of plasma abnormal protein aggregate levels, regional brain volume loss, and poor neuropsychiatric performance. We herein propose that compared with normal controls or PD patients with normal cognition, PD patients with cognitive decline exhibit elevated plasma abnormal protein aggregate levels, lower regional brain volumes, and reduced neuropsychiatric performance. We therefore applied a hypothesis-driven mediation model to determine the possible interactions or casual relationships among elevated plasma abnormal protein aggregate levels, lower regional brain volume, and poor neuropsychiatric performance involved in the cognitive decline of PD patients.

## 2. Methods

### 2.1. Participants

A total of 31 patients with no previous underlying neurological or psychological/psychiatric diseases, taking no neurological or psychotropic medication, were enrolled at the Neurology Outpatient Department in Kaohsiung Chang-Gung Memorial Hospital prospectively. All patients were with no contraindications for receiving magnetic resonance imaging (MRI) and wrote informed consents prior to participation in this study. All patients diagnosed with Parkinson's disease by experienced neurologists according to the Parkinson's Disease Society's criteria [18] in the “OFF” state by evaluating the Unified Parkinson Disease Rating Scale (UPDRS) [19], the Schwab and England (S&E) scale, and modified Hoehn and Yahr staging (H&Y) scale [20]. The UPDRS evaluates part I to IV, including the aspects of mental dysfunction and mood, motor disability, motor impairment, and treatment-related motor and nonmotor complications via clinical observation and interview. The PD patients were further grouped into 2 subgroups according to their cognitive status, including 13 PD with normal cognition (PDN) (7 males and 6 females; mean age: 60.62 ± 7.33 years), and 18 PD with cognitive dysfunction (PDCD) (5 males and 13 females; mean age: 62.89 ± 6.04 years) patients, in accordance with the Movement Disorder Society PD-MCI Task Force diagnostic criteria [21].

An additional 24 age- and gender-matched healthy subjects with no medical history of neurological diseases, brain trauma, psychiatric illnesses, or substance abuse were also enrolled in the control group for comparison. The Local Ethics Committee on Human Research of Kaohsiung Chang Gung Memorial Hospital in Taiwan approves the protocols of this study.

### 2.2. Neuro-Psychological Assessments

All patients received the Wechsler Adult Intelligence Scale-III (WAIS-III) as the neuropsychiatric survey, conducted by a clinical psychologist blinded to the clinical status of each patient, including assessments of attention, executive, speech and language, memory, and visuospatial functions [22]. The digit span test was used to evaluate attention function. The digit symbol coding, similarity, arithmetic, picture arrangement, and matrix reasoning scores were used to evaluate the executive function. The information subtest evaluates the memory function. The vocabulary and comprehension scores assess the speech and language ability of patients, and the picture completion and block design scores are recorded for visuospatial function evaluation.

### 2.3. Plasma Biomarkers

The blood sampling in this study was applied using a 10-ml K3-EDTA tube during the period from 10 : 00 AM to 2 : 00 PM. The centrifugation was performed at 1500-2500 g for 15 minutes with a temperature of 15-25°C by using a swing-out (bucket) rotor. The supernatant from the blood tube was moved to Eppendorf tubes by transferring every 0.5 ml plasma (supernatant) into a 1.5 ml Eppendorf tube. All plasma samples were frozen at -80°C within 3 hours after sampling. The methodology is detailed in our previous studies [9, 10, 23–25].

For *α*-synuclein, A*β*-40, and T-tau measurements, 40 *μ*l plasma was mixed with 80 *μ*l reagent. For NfL and A*β*-42 measurement, 60 *μ*l plasma was mixed with 60 *μ*l reagent. The IMR analyzer (XacPro-S) was used to detect IMR signals to estimate the concentrations of biomarkers according to the concentration-dependent IMR signal. All measurements were applied in duplicate, and the averaged value of the duplicated measurements was recorded as the detected concentration of the biomarker. The standard deviation of the averaged value of the duplicated measurements was calculated and recorded as CV%. The results were accepted only if the CV% was below 20% for all biomarkers. If the CV% was higher than 20%, one more measurement was required if the CV% was higher than 20% and two of the three measurements showing CV% below 20% were recorded as the concentration.

### 2.4. MR Image Acquisition and Data Analysis

The MRI scan protocols and data processing are detailed in our previous study [26]. A VBM analysis based on Diffeomorphic Anatomical Registration Through Exponentiated Lie Algebra (DARTEL) was used for preprocessing and subsequent analyses of T1-weighted structure data. The approach was analyzed using VBM8 toolbox (http://dbm.neuro.uni-jena.de) with the Statistical Parametric Mapping software program (SPM8, Wellcome Institute of Neurology, University College London, UK, http://www.fil.ion.ucl.ac.uk/spm/) implemented in MATLAB R2010a (Mathworks, Natick, MA).

### 2.5. Statistical Analysis

#### 2.5.1. Analysis of Demographic Data, Plasma Abnormal Protein Aggregate Levels, Regional Brain Volume, and Neuropsychiatric Subtest Results

All demographic data were compared among the groups using the univariate analysis of variance test and reported as the mean ± standard deviation. Analysis of covariance was used to analyze differences in plasma abnormal protein aggregate levels with the subject's age and gender as covariates. Analysis of covariance was also used to analyze differences in neuropsychiatric subtest results with the participant's age, gender, and education as covariates. The statistical significance was set at *p* value < .05. All statistical analyses were performed using the SPSS software, version 17, for Windows (SPSS, Chicago, IL, USA).

#### 2.5.2. Voxel-Wise Whole-Brain Gray Matter Volume Comparison

To detect between-group gray matter volume differences, a general linear model which was implemented in SPM8 was used to compare modulated gray matter segments. The statistics are detailed in our previous study [26], and the resultant statistical inferences were considered significant if the cluster level uncorrected *p* value was <.005, with a cluster size of at least 100 voxels. The clusters with significant group main effect were selected for further mediation analysis.

#### 2.5.3. Correlations among Plasma Abnormal Protein Aggregate Levels, Brain Regional Volume, and Neuropsychiatric Subtest Results

Partial correlation analysis was performed to check the relationships among plasma abnormal protein aggregate levels, brain regional volume, and neuropsychiatric subtest results of the PD groups after controlling for age, gender, and education to minimize the potential influences.

#### 2.5.4. Mediation Analysis

To investigate the possible relationships among abnormal protein aggregate levels, brain regional volume, and neuropsychiatric subtest results, a single-level three-variable mediation model was used with applying the PROCESS macro. An accelerated bias-corrected bootstrap test of statistical significance was performed with 5000 bootstrap samples for SPSS [27]. Mediation analysis is used to validate if the direct effect of an independent variable on a dependent variable can be explained by the indirect influence of the mediating variable or not. The statistical significance threshold was defined once the *p* value of the Sobel test was lower than 0.05 for all the relevant paths [28].

## 3. Results

### 3.1. Demographic Data of Participants and the Disease Severity of PD Patients

The demographic data of the participants are shown in Table 1. There were no significant differences among the normal control, PDN, and PDCD groups in terms of age and gender distribution (age: *p* = .489 and gender: *p* = .182). Inspecting the medication, the patient in the PDCD group take medication at high levodopa equivalent daily doses that the patient in the PDN group, because the neurologists tend to provide more medication when a patient showed a cognitive decline. The distribution of the scales in UPDRS I, UPDRS II, UPDRS III, UPDRS 176, H&Y, and S&E is all demonstrated. There was a borderline significant difference in UPDRS II, which primarily involves the motor experiences of daily living. All the patients denied a smoking history. Otherwise, no significant differences were noted among the other scales.

### 3.2. Between-Group Differences in Plasma Abnormal Protein Aggregate Levels

**Figure 1:**
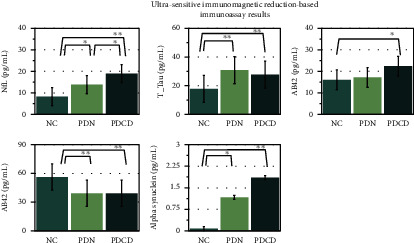
The plasma abnormal protein aggregate levels (pg/mL) of the participants showed that the two PD patient groups had significantly increased plasma levels of NfL, T-Tau, and *α*-synuclein but reduced levels of AB40 compared to normal controls. Furthermore, a significantly higher NfL level was noted in the PDCD group, as compared to both the normal control and the PDN group. ^∗^*p* < .05, ^∗∗^*p* < .001.

The plasma abnormal protein aggregates levels of the participants are listed in Table 1 and Figure 1. Compared to normal controls, the two PD patient groups had significantly increased plasma levels of NfL, T-Tau, and *α*-synuclein but reduced levels of AB40. Of note, only the PDCD group had a significantly increased plasma AB42 level compared with the normal control group.

The two PD patient groups demonstrated elevated plasma NfL levels. More specifically, the PDN patients showed a significantly higher NfL level than the normal controls (mean ± standard deviation; 13.85 ± 5.18 vs. 8.23 ± 4.15; *p* = .003). Furthermore, a significantly higher NfL level was noted in the PDCD group, compared to both the normal control (mean ± standard deviation; 18.92 ± 7.25 vs. 8.23 ± 4.15; *p* < 0.001) and the PDN group (mean ± standard deviation; 18.92 ± 7.25 vs. 13.85 ± 5.18; *p* = .048).

### 3.3. Between-Group Difference in Regional Gray Matter Volume

**Figure 2:**
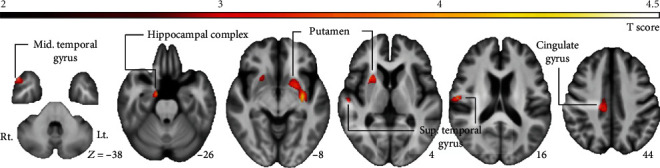
The locations and spatial extents of anatomical regions with significant differences in GMV between the PDN and PDCD groups included bilateral putamen, right hippocampal complex, cingulated gyrus, right superior, and middle temporal gyri.

The locations and spatial extents of anatomical regions with significant differences in GMV between the PDN and PDCD groups are presented in Table 2 and Figure 2. Compared with PDN patients, PDCD patients showed significantly smaller GMVs of the bilateral putamen, right hippocampal complex, right cingulated gyrus, right middle, and superior temporal gyrus.

### 3.4. Correlations among the Affected Brain Regions, Plasma NfL Levels, and the WAIS Subtest Results

The summary of the correlations with their *R* values is listed in Table 3.

#### 3.4.1. Correlation between Affected Brain Regions and Plasma NfL Levels

The plasma NfL level was negatively correlated with the GMV in the right superior temporal gyrus (*r* = −0.462, *p* = 0.012).

#### 3.4.2. Correlation between Affected Brain Regions and the WAIS Subtest Results

The GMV in the left putamen (-32, -12, -11) correlated with the subtest results of Vocabulary, Arithmetics, Matrix reasoning, Digit span, and Letter-number sequencing. The GMV in the right hippocampal complex (14, -7, -26) correlated with the subtest results of Vocabulary, Similarity, Arithmetics, Matrix reasoning, Digit span, Comprehension, and Letter-number sequencing. The GMV in the right putamen (27, 12, 3) correlated with the subtest results of Matrix reasoning, Digit span, and Letter-number sequencing. The GMV in the right superior temporal gyrus (57, -12, 16) correlated with the subtest results of Picture completion, Vocabulary, Digit symbol-coding, Matrix reasoning, and Digit span. The GMV in the right cingulate gyrus (12, -25, 43) correlated with the subtest results of Vocabulary, Similarity, Block design, Matrix reasoning, Digit span, and Letter-number sequencing. The GMV in the right middle temporal gyrus (50, 12, -38) correlated with the subtest results of Matrix reasoning, Digit span, and Letter-number sequencing.

### 3.5. Mediation Analysis for Plasma NfL Level, Certain Affected Brain Region Volumes, and Neuropsychiatric Performance

The primary hypothesis of this analysis was to determine whether the effect of plasma abnormal protein aggregates levels, such as plasma NfL level (independent variable) on neuropsychiatric performance (dependent variable) was explainable directly or indirectly by certain affected brain region volumes (mediator) with a significant group main effect. The path model jointly tested three effects of interest, which are required if the certain affected brain region volume links the plasma abnormal protein aggregates level with the neuropsychiatric performance: (a) the effect of the independent variable (plasma NfL level) on the mediator (the certain affected brain region volume) (indirect effect, path a), (b) the effect of the mediator on the dependent variable (the neuropsychiatric performance) by controlling the effect of the certain affected brain region volume (indirect effect, path b), and (c) the mediation effect a∗b which is defined as the reduction of the relationship between the independent and dependent variables (plasma NfL level—the neuropsychiatric performance) (total relationship, path c) by including the mediator into the model (direct path, path c′).

**Figure 3:**
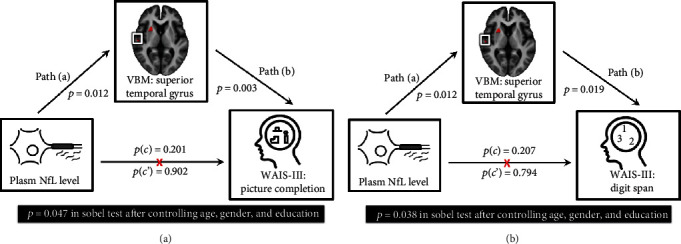
The statistics for mediation models were performed only for the plasma NfL level, the right superior temporal gyrus volume, and the neuropsychiatric subtest results that pass the partial correlation. The mediation relationship models for the plasma NfL level, the right superior temporal gyrus volume, (a) picture completion, and (b) digit span after controlling for age, gender, and education. The two models verifying the poorer neuropsychiatric performances in picture completion and digit span were caused by the vulnerable brain regions loss damaged by the elevated plasma NfL level via a full mediation effect.

For simplicity, we report a full list of results from the present study that fulfill the three criteria cited previously. Consequently, the mediation statistics were performed only for the plasma NfL level, the right superior temporal gyrus volume, and neuropsychiatric subtest, results that passed the partial correlation. The mediation relationship models for picture completion and digit span were significant (*p* = 0.047 and *p* = 0.038 in the Sobel test, respectively) after controlling for age, gender, and education (Figure 3). The two models verifying the poorer neuropsychiatric performances in picture completion and digit span were caused by the vulnerable brain region loss damaged by the elevated plasma NfL level via a full mediation effect.

## 4. Discussion

### 4.1. Summary

Consistent with previous studies and our hypothesis, the PDCD group exhibited higher plasma NfL levels, decreased regional brain volume, and poorer neuropsychiatric subtest results compared with the PDN group. Most of the correlations between these clinical presentations showed significance. The full mediation models among plasma NfL level (independent variable), vulnerable regional brain volume (mediator), and the psychiatric subtest results of picture completion and digit span (dependent variable) were revealed. These results indicate that the vulnerable regional brain volume may be a full mediator in the relationship between plasma abnormal protein aggregate levels and poor psychiatric subtest results in PD patients.

### 4.2. The Origin of Plasma Abnormal Protein Aggregate Levels and Its Source and Influence

Oxidative stress could cause the vulnerable proteostasis network to malfunction, while aging could exacerbate the situation [6]. Once the systemic balance is broken down, the aggregation of abnormal protein fragments accelerates and may be distributed throughout the body, including in blood plasma. Consistent with this, our results demonstrate that plasma abnormal protein aggregate levels can be precisely detected by IMR. It has been reported that higher oxidative stress biomarkers in PD patients are associated with brain microstructure damage and even autonomic dysfunction [17]. The results herein demonstrate that PD patients have higher plasma abnormal protein aggregate levels than normal controls; in addition, PD patients exhibit regional brain atrophy and poor neuropsychiatric scores. The multiple correlations among plasma abnormal protein aggregates, regional brain atrophy, and poor neuropsychiatric performance identified in this study indicate that they are tightly linked, suggesting not only the oxidative stress status of PD patients but also an increased risk of comorbidity or disability.

### 4.3. Correlation of Vulnerable Regional Brain Volume with Plasma Abnormal Protein Aggregate Levels and Psychiatric Subtest Results

This study demonstrates the correlation of the right superior temporal gyrus volume with poor picture completion performance in PD patients. Picture completion ability is associated with visuospatial function, while spatial awareness and spatial neglect associated with the superior temporal gyrus have been shown by studying patients with pure spatial neglect and right brain damage [29]. The superior temporal gyrus is a convergent site of the “what” and “where” aspects of vision, acting as a higher-order area that integrates polymodal sensory inputs. There are multiple connections between the superior temporal gyrus and bilateral frontal lobe regions which affect the planning of purposeful eye and limb movements [30]. This may explain why correlations would exist between the superior temporal gyrus and neuropsychiatric performance, including picture completion, vocabulary, digit symbol encoding, matric reasoning, and digit span. The mediation model of this study indicates not only the picture completion ability of the visuospatial function but also the digit span of the attention function. Taken together, these findings reinforce the key role of the superior temporal gyrus in attention and visuospatial functions.

### 4.4. The Possible Pathophysiology of the Mediation Model

Beyond these associations demonstrated in this study, the establishment of mediation models could provide further statistical evidence to verify and further elucidate the causal relationship [27]. According to our results, a possible pathophysiology might be concluded.

First, as a process of oxidative stress [1], axonal injury, and neuron death in patients with PD, the release of NfL into the surrounding extracellular fluid may reflect the initial damage to the brain microstructure [31]. Subsequently, the released NfL into the microenvironment could lead to ongoing neurodegeneration and neuro-axonal loss in later stages of PD, resulting in brain atrophy [32].

Second, mounting evidence indicates that CSF or plasma NfL levels correlate with brain volume, white matter integrity, or even white matter hyperintensity lesions [31, 33, 34]. Among the whole brain regions, NfL levels could be an independent predictor for hippocampal atrophy [35]. Consistent with previous studies, our results indicate that higher plasma NfL levels correlate with decreased volumes of the hippocampal complex, superior and middle temporal gyri, cingulate, and putamen nuclei in PD patients. Among these regions, interactions between the hippocampal complex and the temporal lobe are of particular interest. Physiologically, it has been proposed that the potential molecular mechanism involved in the hippocampus and temporal cortex is based on proteomics and potentially linked to cognitive status or disorder [36].

Third, from a pathophysiological point of view, oxidative stress could contribute to mesial temporal degeneration [37] and the superior temporal gyrus correlated with the cognitive performance [38]. Our study demonstrates that the volume of the superior temporal gyrus is associated with cognitive performance. More specifically, the vulnerable brain regions affected by oxidative stress experience neuron loss and a tendency to a status of malfunction to dysfunction, thus resulting in poor neuropsychiatric performance.

Finally, as a result of passing the Sobel test in the full-mediation model, it has been suggested that vulnerable regional brain volumes may act as a full mediator in the relationship between plasma abnormal protein aggregate levels and poor psychiatric subtest results in PD patients. Therefore, there would be no direct correlation between plasma abnormal protein aggregate levels and poor psychiatric subtest results if the effects of the vulnerable regional brain volumes were removed. The mediation model investigated in this study indicates that elevated plasma NfL levels caused by oxidative stress and neuro-axonal loss could exacerbate multiple regional brain atrophy and further affect cognitive performance.

## 5. Limitations

Although this study presents a considerable body of evidence, the interpretation of these results should be approached with caution. First, oxidative stress is one cause of plasma abnormal protein aggregate formation; however, there are also influences affecting plasma abnormal protein aggregate levels. Further studies with more oxidative stress biomarkers, like NADPH oxidase 2, hydrogen peroxide, and 8-iso-PGF2*α*, could be conducted to provide more direct evidence of possible pathophysiology. Second, the WAIS-III subtests were used to represent the domains of neuropsychiatric status of PD patients, although this is not the only way to assess cognitive decline in PD patients. Conducting a future study with more neuropsychiatric examinations or specific PD-oriented neuropsychiatric tests could provide more insight. Third, the details related to pathophysiology are extremely complicated, involving temporal relationships and the influence of disease progression, especially given the relatively small sample size of this study. The results of this study provide us with insight into a possible pathophysiology, but not the sole pathogenesis of PD cognitive decline. Finally, unlike a de nova or longitudinal study, confounding factors such as medication, physical status, or comorbidities that may influence the cognitive performance of PD patients cannot be totally ruled out in an observational study.

## 6. Conclusion

This study demonstrates the association between poor neuropsychiatric subtest results, higher plasma abnormal protein aggregate levels, and decreased regional brain volume in PD patients. Our mediation model indicates the possible pathophysiological importance of vulnerable regional brain volumes in the coexistence of plasma abnormal protein aggregates and poor neuropsychiatric subtest performance. This offers valuable insight into the intricate relationship between poor neuropsychiatric subtest performance, elevated plasma abnormal protein aggregate levels, and decreased regional brain volume in PD patients to further clarify the pathogenesis of PD cognitive decline.

## Figures and Tables

**Table 1:** Demographic characteristics of PDN patients, PDCD patients, and control subjects.

	Normal control	PDN	PDCD	*p* value	
				Total	PDN vs. PDCD
Number	24	13	18		
Gender (M : F)	6 : 18	7 : 6	5 : 13	0.182	0.131
Age	60.96 ± 5.10	60.62 ± 7.33	62.89 ± 6.04	0.489	0.302
Education (years)	12.25 ± 0.78	11.84 ± 1.07	8.16 ± 0.91	0.003^∗^	0.012^∗^
UPDRS I		3.46 ± 3.46	3.35 ± 2.84	N/A	0.937
UPDRS II		11.31 ± 9.84	9.41 ± 5.50	N/A	0.045^∗^
UPDRS III		25.92 ± 20.85	22.29 ± 12.84	N/A	0.149
UPDRS 176		40.69 ± 33.24	35.06 ± 19.76	N/A	0.089
Modified Hoehn and Yahr staging		2.15 ± 1.09	1.82 ± 1.02	N/A	0.379
Schwab and England (S&E) scale		79.23 ± 17.06	78.82 ± 23.15	N/A	0.859
Levodopa equivalent daily doses (mg)		257.92 ± 286.40	472.28 ± 250.46	N/A	0.02

Plasma abnormal protein aggregates levels (pg/mL)					
NfL	8.23 ± 4.15	13.85 ± 5.18	18.92 ± 7.25	<0.001^∗^	0.048^∗^
T-tau	17.84 ± 9.44	30.88 ± 12.24	27.71 ± 2.20	0.001^∗^	0.350
AB42	16.08 ± 4.58	17.20 ± 2.74	22.37 ± 1.64	0.035^∗^	0.085
AB40	56.18 ± 13.75	39.23 ± 10.75	39.25 ± 2.91	<0.001^∗^	0.083
Alpha Synuclein	0.07 ± 0.07	1.16 ± 1.03	1.85 ± 0.22	<0.001^∗^	0.052

Wechsler Adult Intelligence Scale-III (WAIS-III)					
Picture completion	10.20 ± 2.66	10.30 ± 2.21	7.38 ± 2.83	0.002^∗^	0.035^∗^
Vocabulary	11.50 ± 2.76	12.15 ± 2.54	8.55 ± 2.25	<0.001^∗^	0.005^∗^
Digit symbol-coding	11.54 ± 3.07	10.15 ± 3.53	6.23 ± 2.46	<0.001^∗^	0.002^∗^
Similarities	10.16 ± 2.76	10.84 ± 2.03	7.44 ± 2.43	<0.001^∗^	0.007^∗^
Block design	9.58 ± 2.99	9.61 ± 2.66	6.16 ± 2.59	<0.001^∗^	0.016^∗^
Arithmetics	11.46 ± 0.48	10.92 ± 0.63	7.22 ± 0.57	<0.001^∗^	<0.001^∗^
Matrix reasoning	9.39 ± 0.64	11.01 ± 0.85	7.74 ± 0.76	0.006^∗^	0.008^∗^
Digitspan	11.34 ± 0.53	11.59 ± 0.71	7.83 ± 0.63	<0.001^∗^	<0.001^∗^
Information	10.46 ± 0.36	10.04 ± 0.48	9.06 ± 0.43	<0.001^∗^	0.147
Picture arrangement	10.14 ± 0.64	10.08 ± 0.86	8.49 ± 0.84	0.007^∗^	0.203
Comprehension	11.75 ± 0.50	11.60 ± 0.67	8.72 ± 0.66	<0.001^∗^	0.005^∗^
Letter-number sequencing	10.37 ± 0.61	11.47 ± 0.84	8.26 ± 0.80	0.037^∗^	0.010^∗^

Age and gender data were compared by ANOVA test. Misfolding protein biomarkers were compared by analysis of covariance (ANCOVA) after controlling for age and gender. Wechsler Adult Intelligence Scale-IV was compared by analysis of covariance (ANCOVA) after controlling for age, gender, and education. Data are presented as mean ± standard deviation. ^∗^*p* < .05.

**Table 2:** Regions showing gray matter volume differences between PD patients with normal cognition (PDN) and cognitive decline (PDCD).

MNI atlas coordinates			Voxel size	Gray matter	BA	GMV (mm^3^)		*t*_max_
X	Y	Z				PDN	PDCD	
-32	-12	-11	753	Putamen, L	—	0.610 ± 0.12	0.483 ± 0.10	4.18
14	-7	-26	108	Hippocampal complex, R	34	0.671 ± 0.12	0.581 ± 0.09	3.56
27	12	3	388	Putamen, R	—	0.721 ± 0.17	0.545 ± 0.15	3.47
57	-12	16	187	Superior temporal gyrus, R	41	0.642 ± 0.16	0.480 ± 0.12	3.43
12	-25	43	178	Cingulate gyrus, R	31	0.856 ± 0.06	0.752 ± 0.11	3.2
50	12	-38	187	Middle temporal gyrus, R	38	0.832 ± 0.11	0.651 ± 0.19	3.16

Abbreviations: MNI: Montreal Neurological Institute; BA: Brodmann area; GMV: gray matter volume; R: right side; L: left side. The statistical criteria were set at joint voxel height uncorrected *p* < .005 and voxel size >100.

**Table 3:** Correlations between neurofilament light chain, certain WAIS subtests, and the specific brain regions of the PD patients.

	Correlation (*r*) of the specific brain regions					
	Putamen, L	Hippocampal complex, R	Putamen, R	Superior temporal gyrus, R	Cingulate gyrus, R	Middle temporal gyrus, R
	(-32, -12, -11)	(14, -7, -26)	(27, 12, 3)	(57, -12, 16)	(12, -25, 43)	(50, 12, -38)
Misfolding protein						
Neurofilament light chain	-0.297	0.052	-0.096	-0.462^∗^	-0.224	0.071

WAIS subtests						
Picture completion	0.176	0.149	0.300	0.581^∗∗^	0.312	0.085
Vocabulary	0.402^∗^	0.517^∗^	0.363	0.382^∗^	0.433^∗^	0.340
Digit symbol-coding	0.298	0.137	0.123	0.421^∗^	0.199	0.129
Similarities	0.305	0.418^∗^	0.358	0.338	0.454^∗^	0.357
Block design	0.353	0.253	0.179	0.315	0.383^∗^	0.173
Arithmetics	0.452^∗^	0.429^∗^	0.273	0.367	0.308	0.325
Matrix reasoning	0.484^∗^	0.440^∗^	0.423^∗^	0.429^∗^	0.394^∗^	0.389^∗^
Digit span	0.621^∗∗^	0.376^∗^	0.466^∗^	0.615^∗∗^	0.420^∗^	0.431^∗^
Comprehension	0.279	0.402^∗^	0.358	0.316	0.300	0.370
Letter-number sequencing	0.525^∗^	0.523^∗^	0.615^∗^	0.329	0.586^∗^	0.660^∗∗^

Correlations between among neurofilament light chain and the specific brain regions of the PD patients were performed by partial correlation after controlling for age, and gender. Correlations between certain WAIS subtests and the specific brain regions of the PD patients were performed by partial correlation after controlling for age, gender, and education. ^∗^*p* < .05, uncorrected. ^∗∗^*p* < .05 with a Bonferroni corrected, accounting for multiple regions of interest and neuropsychiatric subtests comparisons.

## Data Availability

The data that support the findings of this study are available on request from the corresponding author, Meng-Hsiang Chen. The data are not publicly available due to their containing information that could compromise the privacy of research participants.
